# Reduced Fascicle Area Demonstrated in Ilioinguinal Nerves Resected from Primary Inguinal Herniorrhaphy Patients as Evidence of Compression Neuropathy

**DOI:** 10.1155/2024/3339753

**Published:** 2024-05-20

**Authors:** Robert Wright, Donald E. Born, Troy Sanders, Jordan Landes, Troy Salisbury, Anjali S. Kumar, Makena Horne

**Affiliations:** ^1^Elson S. Floyd College of Medicine, Washington State University, 412 E Spokane Falls Blvd, Spokane, WA 99202, USA; ^2^Department of Pathology, Stanford University, 291 Campus Drive, Stanford, CA 94305, USA; ^3^Cascade Hernia and Surgical Solutions at Meridian Surgery Center, 208 17^th^ Ave SE Suite 201, Puyallup, WA 98372, USA; ^4^Loma Linda University School of Medicine, 11175 Campus St. Loma Linda, Loma Linda, CA 92350, USA

## Abstract

**Methods:**

30 male patients with primary inguinal hernias undergoing primary inguinal herniorrhaphy were prospectively recruited for ilioinguinal nerve resection and evaluation. Three samples of the resected ilioinguinal nerve (proximal, canal, and distal) were evaluated using Masson's trichrome stain to measure fascicle and total nerve cross-sectional area and detect changes in collagen.

**Results:**

The fascicle cross-sectional area in the canal segment was significantly decreased compared to the proximal control with a large effect size observed (*p* = 0.016, *η*^2 ^ = 0.16). There was no significant difference in the nerve cross-sectional area between locations, but there was a moderate to large effect size observed between locations (*p* = 0.165, *η*^2 ^ = 0.105). There was no significant difference in collagen content nor effect size observed between locations (*p* = 0.99, *η*^2 ^ = 1.503 × 10^−4^). *Interpretation*. The decrease in the fascicle cross-sectional area within the inguinal canal further suggests that there is chronic pressure applied by hernia tissue consistent with axon degeneration. Collagen content is uniformly distributed along the length of the nerve. Further studies with larger samples are needed to confirm the observed effect of nerve location on the total nerve cross-sectional area and axon loss.

## 1. Introduction

Previous studies have reported apparent enlargement of the nerve distal to the external inguinal ring in as many as 63% primary inguinal hernia patients [1, 2] but have yet to investigate the pathophysiological change resulting in the size discrepancy. Compression neuropathy can result in fibrotic changes to the external epineurium and perineurium, thickening these layers and increasing the overall nerve diameter [3]. Many autopsy series have demonstrated that compression neuropathy commonly occurs at sites where nerves interact with fascia and bone [4, 5], characterized by perineural fibrosis and axon loss with varying degrees of Wallerian degeneration [6, 7]. In animal studies, this has been shown to be a dose-dependent response where increased pressure results in more damage over time [8]. This is thought to be an ischemic damage due to a loss of blood flow in the endoneurium and results in the degeneration of nerves without a significant inflammatory response [9]. Nerves displaying compression neuropathy in an inguinal hernia have been shown to demonstrate an increased diameter adjacent to the compression site, increased fascicle count, and increased myxoid content (edema) [1, 10, 11]. This is due to the nerve becoming constricted and eventually damaged due to nearby structures exerting increased pressure by the hernia sac over a prolonged period [1, 4, 5]. The purpose of this study is to investigate sections of the ilioinguinal nerve from primary inguinal hernia patients at several sites within the inguinal canal for evidence of collagen deposition, axon loss, and changes in the nerve cross-sectional area to further explain gross nerve size discrepancies.

## 2. Materials and Methods

**Figure 1:**
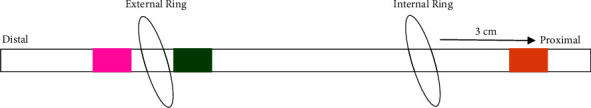
Illustration of the location of the sample site along the ilioinguinal nerve. Proximal control sample (nerve not in contact with hernia tissue) in orange. Inguinal canal segment proximal to external ring in green. Distal segment found distal to the external inguinal ring in pink.

Thirty patients undergoing primary inguinal herniorrhaphy were prospectively recruited and consented for ilioinguinal neurectomy and evaluation. These patients were not part of a previous primary inguinal hernia study. During primary inguinal hernia repair using the Lichtenstein tension-free mesh repair, the ilioinguinal nerve was visually inspected for size discrepancy at the external inguinal ring. Using the best intraoperative judgment, the surgeon resected ilioinguinal nerves which were grossly enlarged just beyond the external ring during this repair. Due to the ilioinguinal nerve's origin proximal to the inguinal canal, an uninvolved site of the ilioinguinal nerve was able to serve as a control to the involved sections of the nerve (Figure 1). Using the resected specimen, three segments of the nerve were selected: one 3 cm proximal to the internal inguinal ring, one in the inguinal canal within 1 cm of the external ring, and one just distal to the external inguinal ring (Figure 1). Specimens were fixed in formalin solution and sent for the permanent section. All measurements of the cross-sectional area were taken postfixation and may be smaller than *in vivo* due to shrinkage associated with formalin fixation. Histological characterization was conducted to quantify the total fascicle cross-sectional area, total nerve cross-sectional area, and collagen content. These measurements were pursued to identify suspected evidence of size discrepancy and collagen content that could be suggestive of compression neuropathy. To best accomplish this task, a trichrome stain was selected. Trichrome stains have a wide variety of applications in muscle fibers and other tissues and can be effectively applied to nerves to differentiate collagen fibers from other structures [12]. The collagen content of the nerves was examined due to its role in fibrosis and thus significance in determining presence of compression neuropathy [1, 7].

**Figure 2:**
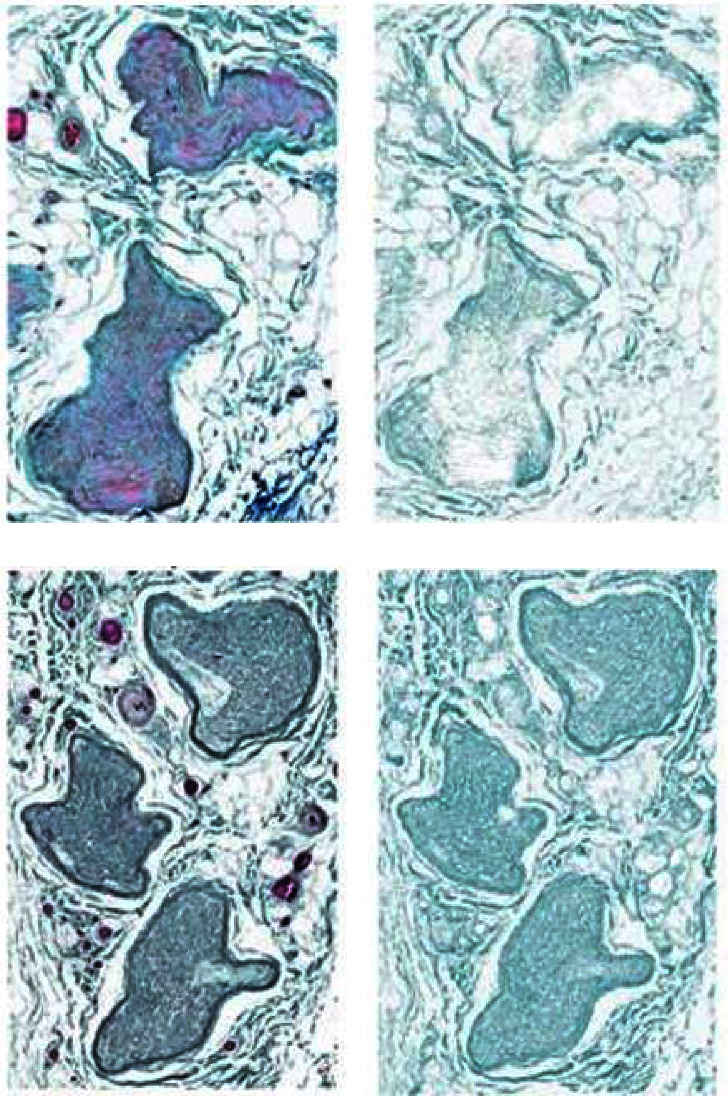
Representative protected and affected ilioinguinal nerve samples stained with trichrome (a, c), next to the resultant color deconvoluted image containing only the green component (b, d), showing increased collagen content in the inguinal canal sample. (a) Proximal sample. (b) Proximal green component. (c) Inguinal sample. (d) Inguinal green component.

After formalin fixation, the segments were paraffin-embedded, oriented, cross-sectioned, and stained with modified Masson's trichrome (collagen green, myelin red, and nuclei dark red-brown) [13]. The stained sections were digitized (Aperio AT2, Leica Biosystems, USA) and deidentified. The samples were then analyzed by a blinded reviewer using Fiji ImageJ (Version 1.52n, NIH, USA). The nerve cross-sectional area (mm^2^) was measured by manually drawing the region of interest (ROI) around the entire nerve (Figure 2). An additional set of ROIs were drawn around the individual nerve fascicles (within the nerve sections, excluding the perineurium). The multiple ROIs selecting the nerve fascicles were then summed to determine the total cross-sectional area covered by nerve fascicle. Using a filter customized to isolate the green wavelength dominant in Masson's trichrome stain, color deconvolution in Fiji was applied to separate the image into the primary green, blue, and red components [5, 6]. To quantify collagen content, the total cross-sectional area (mm^2^) covered by the green component was measured against fascicle ROI total cross-sectional area. The proximal section of the ilioinguinal nerve, unaffected by hernia tissue, was used as the control segment from which to compare the inguinal canal segment and the distal segment. This study has undergone continuous review and approval by our Institutional Review Board.

Three dependent variables (nerve cross-sectional area, fascicle total cross-sectional area, and collagen total cross-sectional area) were measured across the three sites (proximal, canal, and distal.) Statistical analysis was performed using JASP computer software (Version 0.18.3, JASP Team 2024.) Outliers were identified based on the interquartile range for each dependent variable and removed prior to analysis such that acceptable levels of skewness and kurtosis [14] could be achieved. Q-Q plots were also examined after the removal of outliers for each dependent variable at each location to identify potential deviations from normality. It was determined that all dependent variables at all locations met the assumption of normality once outliers were removed from the data. Parametric methods were utilized for all analyses.

Given that three separate ANOVAs will be conducted to address the research questions, there is the possibility for an inflated family-wise error rate. To avoid this, sequential Holm–Sidak adjustment [15] was implemented for the *p* values from each omnibus ANOVA. In this process, *p* values resulting from the analysis are ordered from small-to-large and then adjusted based on the sequential Holm–Sidak formula. This method has the benefit of limiting family-wise type-I error while maintaining higher statistical power than the commonly utilized Bonferroni procedure [15]. Both adjusted and unadjusted *p* values are to be reported. The overall type-I error rate was set to 5% for all analyses (*α*=0.05).

To further assess whether assumptions were met for ANOVA, the dependent variables across sites were examined for sphericity. Results of the analyses suggested that sphericity could not be assumed for the collagen outcome (*p*=0.034), so a Greenhouse–Geisser correction was used when analyzing that dependent variable. Sphericity could be assumed for all other dependent variables.

## 3. Results

**Figure 3:**
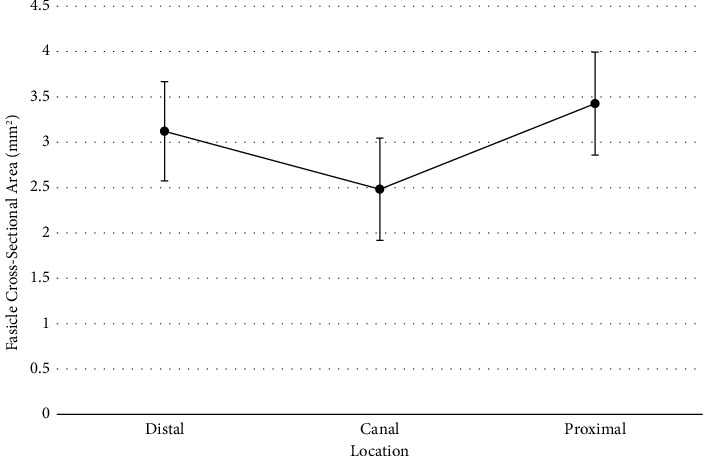
Fascicle area means for each location with 95% confidence intervals.

A total of thirty patients underwent repair and ilioinguinal nerve resection. Patient characteristics are listed in Table 1. Of these resected nerves, the data from four patients were incomplete, resulting in twenty-six complete sample sets. To determine if differences existed for the fascicle cross-sectional area depending on proximal, canal, or distal location, a repeated measures ANOVA was conducted. Repeated measures analysis was used due to all measures coming from the same participants, and the assumption of sphericity was met according to Mauchly's test of sphericity (*p*=0.271). Results of the analysis did not reveal a statistically significant difference for the fascicle cross-sectional area between all three locations (*F*(2, 46)=4.46, Holm − Sidak *p*=0.050, *η*^2^=0.16). However, the effect size observed would be considered large according to Cohen's rules of thumb [16]. The effect size suggests that 16% of the variance in the fascicle cross-sectional area could be explained by which location was measured. A larger sample would be needed to confirm the observed effect, and the result was very close to statistical significance even after adjusting for multiple testing. Given that a meaningful effect was found, *Holm* post hoc comparisons were conducted to see where potential differences could be found. A statistically significant, moderate-to-large difference was found between canal and proximal locations (*p*=0.016, *d*=−0.67 [95% CI : −1.29, −0.05]). The effect size for this difference suggests that the proximal fascicle cross-sectional area was over 0.67 standard deviations higher than the canal fascicle cross-sectional area. Although nonsignificant, a moderate effect size difference was found between canal and distal locations (*p*=0.107, *d*=−0.46 [95% CI : −1.05, 0.136]). Distal and proximal locations only showed a small effect size difference that was not statistically significant (*p*=0.35, *d*=−0.22[95% CI : −0.79, 0.357]) (Figure 3).

**Figure 4:**
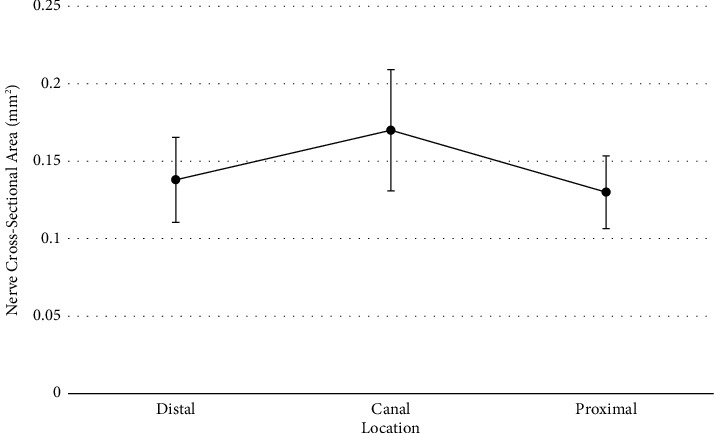
Nerve cross-sectional area means for each location with 95% confidence intervals.

To determine if differences existed for the nerve cross-sectional area depending on proximal, canal, or distal location, a repeated measures ANOVA was conducted. Repeated measures analysis was used due to all measures coming from the same participants, and the assumption of sphericity was met according to Mauchly's test of sphericity (*p*=0.539). Results of the analysis did not reveal a statistically significant difference for the nerve cross-sectional area between the locations (*F*(2, 44)=2.59, Holm − Sidak *p*=0.165, *η*^2^=0.105). However, the effect size observed would be considered moderate to large according to Cohen's rules of thumb [16]. The effect size suggests that almost 11% of the variance in the nerve cross-sectional area could be explained by which location was measured. A larger sample would be needed to confirm the observed effect. Given that a meaningful effect was found, *Holm* post hoc comparisons were conducted to see where potential differences could be found. Although no statistically significant differences were found, a small-to-moderate effect size difference was found between canal and distal (*p*=0.19, *d*=0.42 [95% CI : −0.21, 1.05]), and a moderate effect size difference was found between canal and proximal (*p*=0.109, *d*=0.53 [95% CI : −0.11, 1.17]). Average nerve cross-sectional areas were 0.42 standard deviations apart for canal and distal locations and 0.53 standard deviations apart for canal and proximal locations (Figure 4).

**Figure 5:**
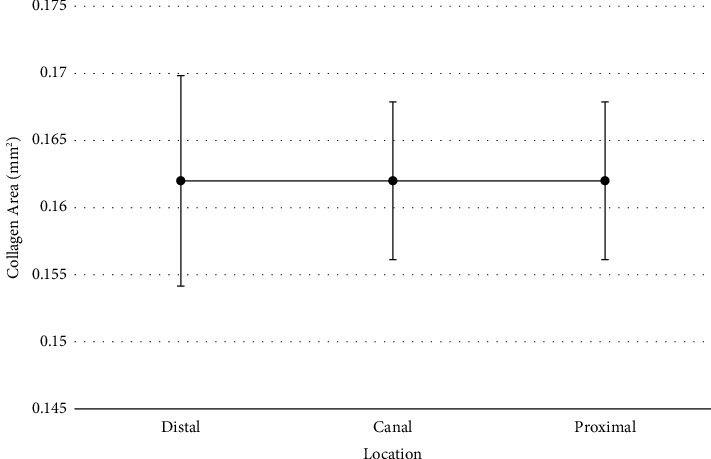
Collagen area means for each location with 95% confidence intervals.

To determine if differences existed for the collagen cross-sectional area depending on proximal, canal, or distal location, a repeated measures ANOVA was conducted. Repeated measures analysis was used due to all measures coming from the same participants, but the assumption of sphericity was not met according to Mauchly's test of sphericity (*p*=0.034). The ANOVA results were adjusted using a Greenhouse–Geisser correction. Results of the analysis did not reveal a statistically significant difference for the collagen cross-sectional area between the locations (*F*(1.58, 36.37)=0.003, Holm − Sidak *p*=0.99, *η*^2^ < 0.01). No post hoc comparisons were conducted since no overall mean differences existed (Figure 5). This suggests that collagen content appears to be uniform at each segment of the nerve. Statistics are summarized in Table 2.

## 4. Discussion

Prior research on size disparity of nerves in the inguinal canal has demonstrated significant correlation with preoperative hernia pain. This current study demonstrated a decrease in the fascicle cross-sectional area in the canal region of the ilioinguinal nerve compared to the proximal control which suggests compression damage to the nerve from the hernia. This could be indicative of axon loss in the canal region of the ilioinguinal nerve. Additionally, this study found similar cross-sectional areas between the proximal and distal regions of the nerve suggesting that damage is occurring within the canal, and the nerve may be narrowing in the canal region rather than enlarging at the distal region. Apparent decrease in the size of the nerve in the inguinal canal correlates with preoperative pain “most of the time” on questionnaire series. Additionally, it correlates with heavy fibrosis of the external inguinal ring as noted during surgery [10]. In another study, decreased canal segment diameter visually noted during surgery correlates with four of eight pain measures on Carolina's Comfort Scale for preoperative pain [2].

It was originally hypothesized for this study that collagen was depositing in the nerve, accounting for nerve enlargement, but this study did not find unequal collagen deposition in the different locations (Table 2). However, in yet another study of nerve, decreased canal diameter correlated with increased pain as well as increased evidence of myxoid material which also correlated with pain [1]. The reduced fascicle area within the canal region of the nerve could be indicative of axon loss. Demyelination and axonal degradation have been found in chronic compression studies of rat sciatic and sural nerves. Increased pressure on the nerve can create ischemic changes that result in nerve fiber degeneration [5] which is consistent with the findings of this study and further suggestive of compression neuropathy. Masson's trichrome stain has limited ability to study axon loss, further research with Toluidine blue could be considered to better visualize demyelination and the severity of axon loss within nerves and conclude the state of nerve injury. These studies suggest that the hernia itself is causing nerve compression neuropathy and intervention prior to pain may prevent the progression of compression neuropathy. It is well established that the degree of preoperative pain when in higher levels correlates with chronic postoperative pain [17], so prevention of preoperative pain is a worthy goal. It is also a mystery as to why so many postoperative hernia patients have pain by whatever approach the repair is done. The key may lie in compression neuropathy occurrence.

Currently, there is an acceptable “watch and wait” approach to inguinal hernia repair. Patients are routinely told that it is advisable to delay treatment related to their hernia until it is bothering them more due to increased pain. This is based on the prospective data demonstrating that the yearly risk of acute strangulation of inguinal hernias is about 1–3% [18]. However, if the hernia sac is exhibiting a chronic pressure on the ilioinguinal nerve, it is likely contributing to the degeneration of nerve fibers. This challenges the current “watch and wait” approach to avoid further nerve damage.

### 4.1. Limitations

This study does not have large numbers and is a single-surgeon series. The moderate-to-large effect sizes seen in the analyses of total nerve and fascicle cross-sectional areas indicate that there may be a variance in cross-sectional areas by location, and larger studies would help investigate this relationship further. Tissue shrinkage commonly known to occur with formalin fixation may have made previously visible size discrepancies less noticeable and may have affected the specimens microscopically and not uniformly. The measurements are performed by one researcher without intraobserver repeatability testing. Also, to study the ilioinguinal nerve, it must be sacrificed during herniorrhaphy which is not advised as a standard practice. If compression neuropathy is to be established as a real entity in patients with primary inguinal hernias, larger funded studies will need to be performed which include stains that can demonstrate axon loss and Wallerian degeneration.

## 5. Conclusion

In patients with a primary inguinal hernia who present with a visible size discrepancy between the ilioinguinal nerve in the inguinal canal and the same nerve at the external inguinal ring, the smaller canal segment may be a result of compression neuropathy. This is supported by the findings of the decreased cross-sectional area of the nerve fascicle in the inguinal canal compared to the proximal nerve. Collagen deposition is uniformly distributed, so the size difference in the canal segment fascicle may be due to axon loss which is common in compression neuropathy.

## Figures and Tables

**Table 1:** Patient characteristics.

	*N* ^*∗*^	Mean	Std. dev	Range
Age	29	57	14.3	27–87
BMI	29	28.3	4.1	21–38
		Right side	Left side	
Hernia laterality	29	13	16	
		Yes	No	
Incarcerated?	29	21	8	
		<1.5 cm	<3 cm	>3 cm
Hernia type-direct	23	13	9	1
Hernia type-indirect	6	4	2	

^*∗*^Of the original thirty patients, one patient's dataset was incomplete.

**Table 2:** Measurements of the total nerve cross-sectional area, total fascicle cross-sectional area, and total collagen cross-sectional area at the three sample sites.

Sample location	*N* ^*∗*^	Proximal (control)	Canal	Distal	Canal vs. proximal	Canal vs. distal	Proximal vs. distal
		Mean (mm^2^)	Mean (mm^2^)	Mean (mm^2^)	*p*	*p*	*p*
Total nerve cross-sectional area	23	0.130	0.170	0.138	0.109	0.190	0.655
Total fascicle cross-sectional area	24	3.426	2.482	3.121	**0.016**	0.107	0.350
Total collagen cross-sectional area	24	0.162	0.162	0.162	1	1	1

^*∗*^ *N* is calculated with outliers removed; see Materials and Methods section. Numbers in bold indicate significance.

## Data Availability

The data that support the findings of this study are available from the corresponding author under reasonable request.
